# An integrated algorithm for single lead electrocardiogram signal analysis using deep learning with 12-lead data

**DOI:** 10.1038/s41598-025-18910-1

**Published:** 2025-10-07

**Authors:** Muhammad Farhan Safdar, Robert Marek Nowak, Piotr Pałka, Ahmed Al Faresi

**Affiliations:** 1 Faculty of Electronics and Information Technology, Warsaw University of Technology, Warsaw, Poland; 2 College of Information Technology, United Arab Emirates University, Al Ain, UAE

**Keywords:** Electrocardiogram, Deep learning, Neural network, Healthcare wearable technology, Diagnostic signals, Information technology, Computer science

## Abstract

Artificial intelligence (AI) algorithms have demonstrated remarkable efficiency in analyzing 12-lead clinical electrocardiogram (ECG) signals. This has sparked interest in leveraging cost-effective and user-friendly smart devices based on single-lead ECG (SL-ECG) for diagnosing heart dysfunction. However, the development of reliable AI model is influenced by the limited availability of publicly accessible SL-ECG datasets. To address this challenge, presented study introduces a novel approach that utilizes 12-lead clinical ECG datasets to bridge this gap. We propose a hierarchical model architecture designed to translate SL-ECG data while maintaining compatibility with 12-lead signals, ensuring a more reliable framework for AI-driven diagnostics. The proposed sequential model utilizes a convolutional neural network enhanced with three integrated translational layers, trained on individual 12-lead clinical ECG, to significantly improve classification performance on SL-ECG. The experimental analysis is conducted using three benchmark datasets: Physikalisch-Technische Bundesanstalt (PTB-XL), Computing in Cardiology Challenge 2017, and China Physiological Signal Challenge 2018. This study also evaluates the effects of denoising techniques and lead polarity variations, including biphasic and negative deflections. Results show that the model achieved over 82% test accuracy on unseen SL-ECG signals, with an area under the receiver operating characteristic of 0.81, sensitivity of 76.60%, and specificity of 83.44% when trained on clinical lead I. Additionally, leads II, V4, and V5 demonstrated potential for effective AI model training. The work supports advancement of smart devices by enhancing SL-ECG classification and assists clinicians in assessing heart abnormalities more effectively.

## Introduction

An electrocardiogram (ECG) records the heart’s electrical activity and was first measured by A.D. Waller using Lippmann’s inventions in 1887 ^1^. However, factors such as inertia and friction among capillaries, which were not considered by early scientists, led to imperfect measurements. These issues were later addressed by Willem Einthoven in 1901 ^2^. Over time, significant advancements have been made, leading to the modern era, where single-lead electrocardiograms (SL-ECG) from smartwatches, wristbands, and smart devices, such as AliveKardia are increasingly in demand for preliminary cardiac diagnosis ^3,4^.

A single-lead ECG recorded using a portable or pocket-sized device can be taken anywhere without medical staff intervention, and the results can be shared with a healthcare practitioner. Smart device-generated ECG signals are approved by the FDA (Food and Drug Administration) and CE-marked (Conformité Européenne) for use with caution. These devices provide about 30-seconds ECG recording for preliminary atrial fibrillation detection ^5^. Atrial fibrillation is a type of arrhythmia and is one of the most significant triggers of ischemic stroke in individuals with irregular heart rhythms. Extending the duration of ECG recordings, such as those of 30 seconds or more, can increase the likelihood of detecting atrial fibrillation ^6^. The 12-lead ECG, commonly referred to as a clinical ECG, provides additional information about heart conditions. However, unlike single-lead ECG, it requires healthcare professionals for administration and is not practical for self-use by patients.

**Figure 1:**
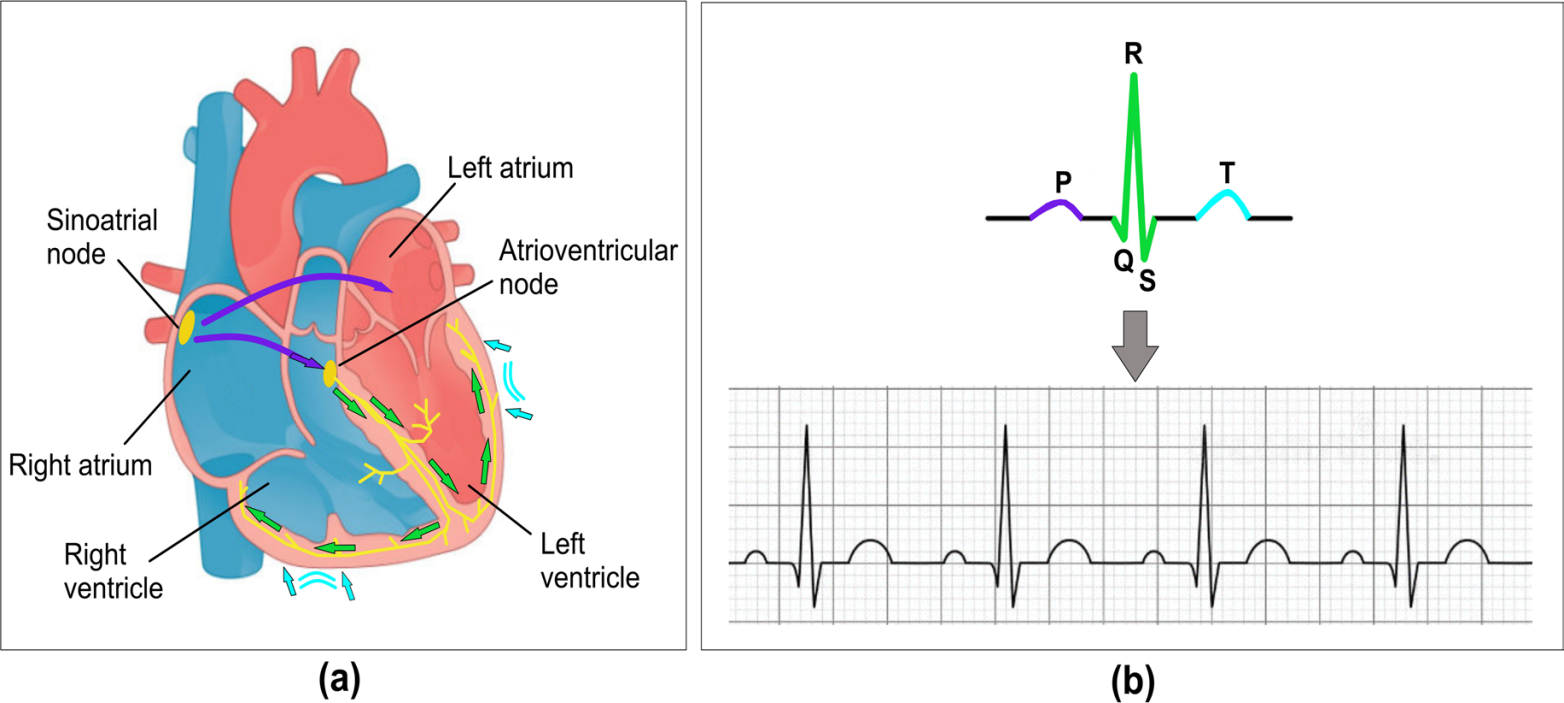
The concept of ECG measurement is based on the electrical impulses generated within the human heart. (**a**) The heart, consisting of atria and ventricles, where yellow lines represent the transmission of electrical pulses to different muscle regions, initiating the depolarization and repolarization processes. (**b**) A single cardiac cycle depicted through electrical stimulation and a corresponding typical ECG waveform.

### Heart cycle morphology

In Fig. 1(a), the electrical impulse originates from the sinoatrial (SA) node, often referred to as the heart’s natural pacemaker. This impulse spreads across the right and left atria, reaching the atrioventricular (AV) node, which generates the 'P' wave. A brief delay follows, commonly observed as the P-Q segment, represented as a straight line in Fig.1(b). After this delay, the impulse travels through the bundle of His and along the left and right bundle branches, stimulating the Purkinje fibers. This activation causes the ventricles to contract, forming the 'QRS' complex, depicted in green within the single cardiac cycle. As the impulse propagates through the ventricles, another straight-line segment, known as the 'ST' segment, appears. At this stage, both ventricles contract to pump blood: the right ventricle sends deoxygenated blood to the lungs via the pulmonary valve, while the left ventricle pumps oxygenated blood to the body through the aortic valve. As the ventricles relax, repolarization occurs, generating the 'T' wave, which is illustrated in purple beneath the left and right ventricles ^7^.

### Measurement of single lead ECG

The SL-ECG follows the lead-I morphology of the 12-lead ECG, where the negative electrode is placed on the right arm and the positive on the left, forming Einthoven’s triangle Lead I ^8^. Since bodily fluids contain ions that conduct electricity, the heart’s electrical activity can be detected non-invasively on the skin. Measuring these signals requires conductive electrodes.

The AliveCor Kardia mobile device features two stainless steel electrodes positioned between the left and right arms, where the user places their fingers. These electrodes capture electrical signals, but low amplitudes may disturbing key features. To enhance visibility, the device applies pre-processing steps such as amplitude adjustment, noise reduction, and filtering. It connects to a smartphone via Bluetooth, transmitting ECG data for display. A minimum 30-seconds recording is typically required to apply these pre-processing methods before presenting the results on the patient’s smartphone screen ^9,10^.

Similarly, the Apple Watch (Series 4) is equipped with stainless steel electrodes, one on the side (digital crown) and another on the underside of the watch. Wearing the watch on the left wrist and placing a finger from the right hand on the digital crown establishes a lead-I morphology, similar to clinical lead I in Einthoven’s triangle. The electrodes capture electrical signals and transmit them to the device’s algorithm for pre-processing. Results are displayed in real-time on the watch screen and can be shared with a smartphone. The signal acquisition and processing algorithms are highly optimized and developed by the manufacturer ^9–11^.

The single-lead ECG requires minimal equipment, unlike the 12-lead ECG, which involves multiple cables and electrodes. Moreover, it can be performed by the patient at home or in any setting. Nevertheless, SL-ECG, in its current state, is not a replacement for a 12-lead clinical ECG, which provides more comprehensive information. For instance, a depressed ST segment observed in leads V5–V6 is crucial for detecting atrial fibrillation ^7^.

### Study significance

Deep learning (DL), a subfield of artificial intelligence (AI) and machine learning (ML), requires a large volume of labeled data for model training in disease detection. Given this, several publicly available 12-lead ECG datasets are hosted on the well-known PhysioNet repository ^12^. This study leverages existing PhysioNet resources and makes the following contributions:

- Investigate and address the challenges in comparing clinical 12-lead ECG with SL-ECG, highlighting key differences and limitations that impact disease detection performance.
- Propose a novel model (algorithm 2 and 3) that predicts diseases from single-lead ECG, trained using 12-lead clinical ECG datasets such as PTB-XL and CPSC-2018, overcoming the challenge of limited single-lead ECG data.
- Identify and determine the most reliable isolated leads from clinical 12-lead ECG for training deep learning models, optimizing performance for disease prediction from SL-ECG.
- Develop a segmentation approach (algorithm 2) for handling prolonged SL-ECG signals, addressing memory overhead challenges and enabling the illustration of all relevant features for accurate diagnosis.

This work is organized as follows: In Section 2, a comprehensive review of the relevant literature on clinical and SL-ECG is presented. Section 3 outlines the methodology, including a novel deep learning model architecture, additional layers, algorithms, and a description of the data. Section 4 presents the experimental setup, results, and a detailed discussion. Finally, Section 5 concludes the work and summarizes the key findings.

## Related Work

Deep learning architectures, with certain modifications, have been extensively utilized for classifying diseases from 12-lead ECG signals using pre-processed images or time-series data. A related study by Raymond A. et al. ^13^ employed a 16-layer VGGNet model pre-trained on ImageNet weights. The concept of using a pre-trained model aligns with transfer learning, where a model trained on one dataset is adapted for another; however, using data from a similar domain is recommended. The study performed binary classification between multiple diseases and normal cases. Data was sourced from PTB-XL, CPSC-2018, and two research papers, with training and testing conducted on 10-12-second-long signals. The model achieved the highest performance in detecting atrial fibrillation, reaching approximately 0.99 AUROC. In another study, Fahad K. et al. ^14^ utilized a 1D deep learning-based residual network (ResNet) to classify five heartbeat types from 12-lead ECG signals in the MIT-BIH dataset. Since time-series data was used instead of images, a sample enhancement technique, the Synthetic Minority Oversampling Technique (SMOTE), was applied. Results from 10-fold cross-validation demonstrated 98.63% accuracy and an F1-score of 92.63%, highlighting the effectiveness of the proposed model.

Recently, hybrid models, which combine two different architectures, have been introduced to enhance classification performance, as highlighted in our prior review-based study ^15^. A similar approach was adopted by Eleyan A. et al. ^16^, who utilized a combination of convolutional neural networks (CNN) and long short-term memory (LSTM) networks to classify heartbeats and congestive heart failure from 12-lead ECG signals. Their study used the MIT-BIH and BIDMC datasets, incorporating fast Fourier transformation (FFT) as a pre-processing technique. The proposed hybrid model demonstrated high classification performance, achieving accuracy, precision, and an F1-score of approximately 99%. Furthermore, ECG classification can also be performed by segmenting heartbeats using delineation techniques, where key ECG features such as the P-wave, QRS complex, and T-wave are identified and extracted. A related study by Annisa D. et al. ^17^ applied this approach to 12-lead ECG signals for beat segmentation using the Lobachevsky University Database (LUDB) dataset. Their work focused on pre-processing techniques, including noise removal and a hybrid CNN-LSTM model, where CNN was used for feature extraction and LSTM for classification. The proposed method achieved an accuracy of 98.82% and an F1-score of 95.93% across approximately 14,588 beats.

Smart devices, including smartwatches, have become increasingly prevalent, as discussed in section 1. A study by Xiangyu Z. et al. ^18^ utilized the Computing in Cardiology (CinC) 2017 dataset, which contains ECG recordings ranging from 30 to 60 seconds, for the classification of atrial fibrillation (AFib), other arrhythmias, normal rhythms, and excessively noisy signals. The authors evaluated a hybrid approach combining a temporal convolutional network (TCN) and ResNet for classification. TCN, a convolutional model variant designed specifically for time-series data, was implemented as a one-dimensional fully convolutional network with dilated causal convolutions, demonstrating superior performance. The proposed model achieved an accuracy of 97% with an F1-score of 87%. However, it is important to note that both the training and testing data, comprising single-channel ECG recordings, were collected from the same device.

A widely used alternative for remote cardiac monitoring in hospitals is the Holter monitor. Luongo G. et al. ^19^ investigated a dataset collected from a Holter device, consisting of 10,234 five-minute ECG recordings obtained from 26 AFib patients and 26 healthy individuals. Instead of deep learning, the authors employed a simple ML approach, specifically a decision tree classifier, trained and tested using a 5-fold cross-validation strategy. The study focused on beat-to-beat interval analysis, achieving an accuracy of 73.5%, a specificity of 91.4%, and a sensitivity of 64.7%.

Jiwoong K. et al. ^20^ investigated the identification of AFib from normal sinus rhythm using self-collected ECG data over a two-year period with a mobile-based mobiCARE-MC100 device. Their dataset comprised 13,509 ECG recordings from 6,719 individuals, with a significant class imbalance–10,287 normal samples compared to fewer AFib cases. To address this imbalance, they applied a random under-sampling technique. The study evaluated recurrent neural networks (RNN), long short-term memory (LSTM), and ResNet50 models, with ResNet50 yielding the highest performance: 70.5% accuracy, an F1-score of 71.9%, 65.8% precision, 79.3% recall, and an AUROC of 0.79. The original challenge for the CinC-2017 dataset, hosted on the PhysioNet repository, was tackled by Datta S. et al. ^21^, who secured the highest recorded score with an overall F1-score of 0.83. Their work, later published at the CinC-2017 conference, employed more than 8,500 ECG recordings for binary classification, distinguishing SL-ECG signals as normal, AFib, or other arrhythmias. The study incorporated extensive pre-processing techniques, including noise removal, PQRST detection, and Fourier transformation. For classification, they implemented ML-based models, specifically adaptive boosting (AdaBoost), achieving an F1-score of 0.86 on the hidden test set for AFib detection.

The classification of diseases using either 12-lead or single-lead ECG has been extensively studied, demonstrating remarkable success. However, utilizing 12-lead ECG data to classify conditions from single-lead ECG remains a challenge and presents opportunities for further improvement. Khunte A. et al. ^22^ isolated lead I from 12-lead ECG and augmented it with noise from MIT-BIH and KardiaMobile 6L device signals. They trained and tested a 7-layer CNN model, which achieved an AUROC of 0.72 for the standard model and 0.87 for the noise-adapted model in detecting left ventricular systolic dysfunction (LVSD). However, it remains unclear whether their testing dataset consisted solely of lead I isolated from 12-lead ECG signals or included SL-ECG data directly from wearable devices. Furthermore, their approach of adding noise for data augmentation raises concerns about potential biases in analysis, as noise removal is a critical step in real-world signal processing.

Similarly, Attia Z. et al. ^23^ predicted LVSD in relation to low ejection fraction (EF) using SL-ECG signals from a digital stethoscope. Their study included 100 patients, of whom only 14 had an EF below 50%, leading to a disproportionately high normal-to-abnormal ratio (86:14). The SL-ECG signals were recorded from four distinct chest locations and compared to 12-lead ECG signals. Among these, the V2 position demonstrated the highest predictive performance, achieving an average AUC of 0.88 in patients with EF < 50%.

**Table 1:** Summary of methodologies, key features, and limitations in ECG based AI models.

Study	Data	Model	Key Features	Limitations
Raymond et al.^13^	PTB-XL, CPSC-2018	VGGNet-16	Utilized identical way of transfer learning with ImageNet weights.	$\bullet$ Predictions were based solely on 12-lead ECG.$\bullet$ Model complexity due to high parameter count.
Eleyan A. et al.^16^	MIT-BIH, BIDMC	CNN-LSTM	A hybrid model incorporating Fourier transformation was used for heartbeat classification.	$\bullet$ Increased computational complexity due to hybrid architecture. $\bullet$ Single-lead ECG (SL-ECG) was not considered for predictions.
Annisa D. et al.^17^	Lobachevsky University database	CNN-LSTM	CNN was employed for feature extraction, while LSTM handled classification in a hybrid approach.	$\bullet$ Computational overhead remains a concern. $\bullet$ Training and testing were performed on identical datasets, limiting SL-ECG prediction.
Xiangyu Z. et al.^18^	CinC-2017	TCN, ResNet	Temporal Convolutional Network (TCN) was evaluated for time-series ECG analysis.	$\bullet$ ResNet, used within the model, adds architectural complexity. $\bullet$ Training and testing relied on the same SL-ECG dataset, demanding high SL-ECG data acquisition.
Jiwoong K. et al.^20^	Private dataset	RNN, LSTM, ResNet-50	Self-collected SL-ECG dataset was used for model development.	$\bullet$ High computational demands for the considered models. $\bullet$ Training and testing were performed exclusively on SL-ECG data only. $\bullet$ New data collection is resource-intensive and time-consuming.
Attia Z. et al.^23^	Self Collection	CNN	A distinct heart condition was predicted using both 12-lead and SL-ECG data.	$\bullet$ Lack of evaluation on widely used wearable SL-ECG devices. $\bullet$ Insufficient analysis of differences between 12-lead and SL-ECG data discrepancies. $\bullet$ Model sustainability regarding artifact removal and segmentation are lacking.

The existing studies and Table 1 indicate that SL-ECG is somewhat comparable to clinical ECG and can be a viable tool for cardiac disease prediction based on AUROC performance ^21^. However, a key challenge arises in model training due to the limited public availability of suitable ECG datasets. To address this gap, the proposed study aims to evaluate the feasibility of training models on clinical 12-lead ECG and predicting diseases using SL-ECG from smart devices. This approach leverages existing resources while mitigating disparities between different datasets.

## Methodology

The proposed study introduces a structured methodology for dataset preparation, innovative DL model architecture design, and optimized environment configuration to enable binary classification across diverse experimental setups. The workflow is outlined as follows:

### Database description

In this study, three datasets were strategically selected for experiments: two clinical 12-lead ECG datasets and one SL-ECG dataset. The PTB-XL ^12^ and CPSC-2018 ^24^ datasets provide 12-lead ECG signals encompassing both pathological and normal samples. PTB-XL comprises approximately 21,000 samples, covering various cardiac conditions such as Conduction Disturbance (CD), Hypertrophy (HYP), Myocardial Infarction (MI), ST/T Change (STTC), and Normal cases, though atrial fibrillation (AFib) samples are relatively low. To ensure a balanced binary classification (normal vs. AFib), AFib samples were extracted from CPSC-2018, which offers a more substantial presence of AFib cases. Specifically, around 1,000 AFib samples from CPSC-2018, varied in interval, i.e., 30 to 60 seconds at a 500 Hz sampling rate, were utilized. Meanwhile, PTB-XL samples, with a 10-second duration and 100 Hz sampling rate, contributed to the dataset composition, ensuring a representative mix for model training. To standardize the data duration and enhance the sample size, the AFib recordings from CPSC-2018 were segmented into 10-second intervals with resampling rate, increasing the total number of AFib samples to 1,669. This segmentation process ensured consistency in signal length across datasets, facilitating more effective model training and comparison. The PTB-XL dataset emphasizes diversity and the presence of co-occurring diseases among its participants ^12^. Similarly, the CPSC-2018 dataset offers a broad and diverse collection, compiled from 11 different hospitals, with many patients exhibiting multiple cardiac conditions ^24^. In contrast, the CinC-2017 dataset consists of single-lead ECG recordings collected and provided by the AliveCor company, designed to reflect real-world testing scenarios using modern wearable ECG device ^25^.

To address the class imbalance between AFib and normal samples in the 12-lead dataset, we chose to augment the AFib class by incorporating AFib samples from both the PTB-XL and CPSC-2018 datasets, as well as miscellaneous disease samples from PTB-XL. This augmentation expanded the AFib class to approximately 10,000 samples. Additionally, we sought to investigate whether the CNN model could learn to recognize the disease from these miscellaneous disease samples, enabling the model to effectively distinguish between normal and diseased samples.

The external test set consisted of single-lead ECG signals from the CinC-2017 dataset ^25^, which were obtained using the AliveCor mobile device with a sampling rate of 300 Hz. These signals had a duration of approximately 30 seconds. To ensure uniformity across datasets and enhance the performance of the deep learning model, the data was down-sampled using the scipy resample library to 100 Hz, matching the sampling rate of the PTB-XL training dataset. Since the CinC-2017 dataset was designed for testing purposes, the unseen validation set, as defined by the dataset owners, was used to evaluate the proposed model’s performance.

### Pre-processing

Denoising is an essential step in ECG signal preprocessing, as raw ECG data often contains various types of noise, such as power-line interference, muscle artifacts, and baseline wander. These unwanted disturbances can develop uncertainty among important features in the ECG waveform, particularly in the presence of arrhythmia’s like AFib. Discrete wavelet transformation (as shown in Algorithm 1) is particularly effective in ECG denoising due to its ability to capture both low-frequency baseline fluctuations and high-frequency noise in different scales. This multi resolution approach allows for precise filtering without losing critical features of the ECG signal, which is essential for accurate classification and analysis. The denoising procedure was applied across all datasets, ensuring that both the 12-lead signals from PTB-XL, CPSC-2018 and the single-lead signals from CinC-2017 were processed consistently and effectively.

**Algorithm 1:**
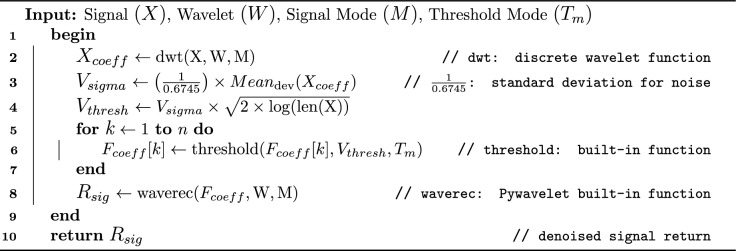
Denoising ECG Signal

**Figure 2:**
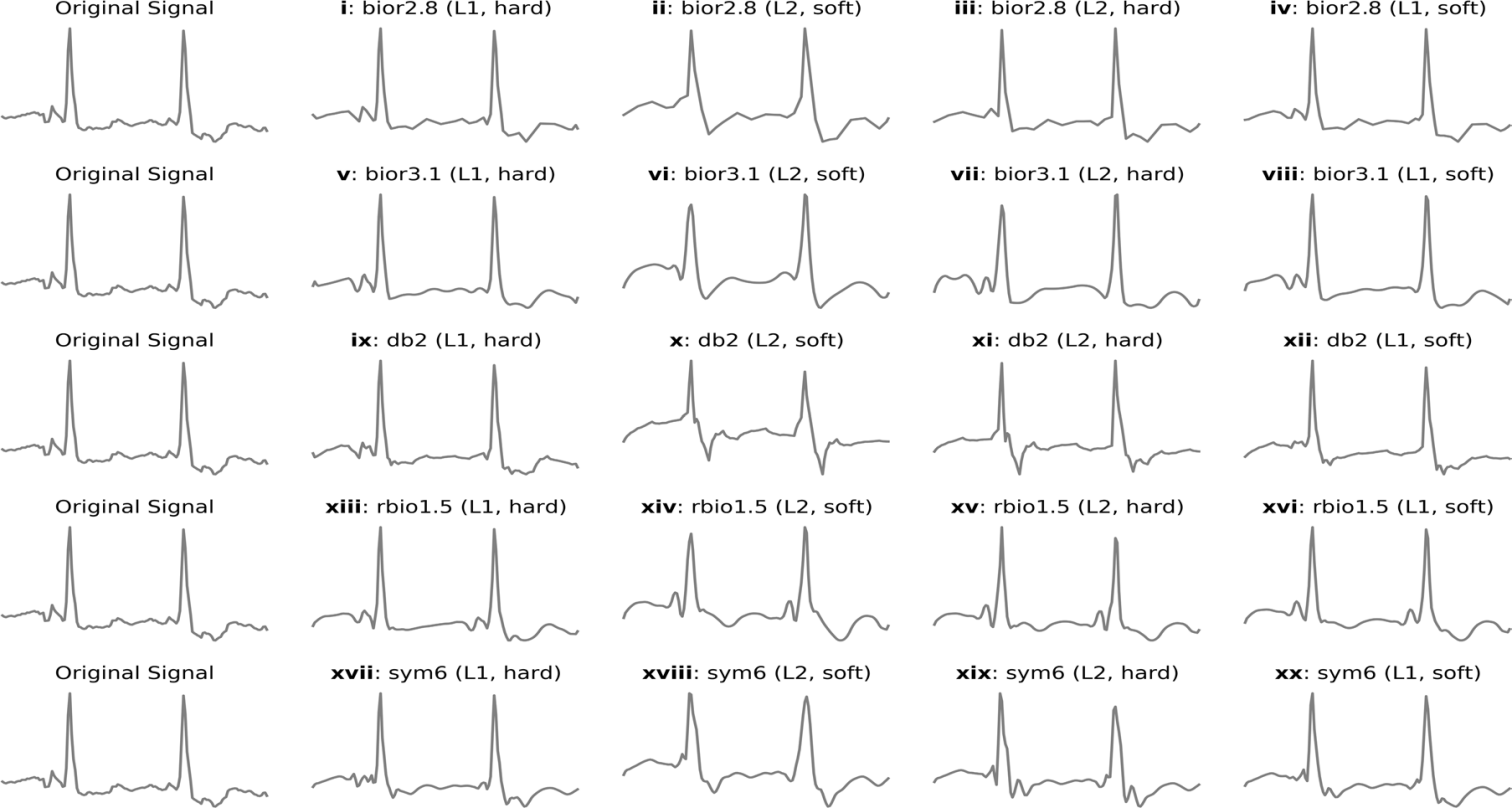
A segmented signal with two heartbeats, illustrating the impact of different wavelets and hyperparameter settings on denoising, with minimal alteration to the original signal.

In the given Algorithm 1, a standard and efficient signal denoising procedure ^26^ with the function discrete wavelet transform (dwt) was adopted that takes three parameters: signal, type of wavelet as 'bior3.1' and mode for boundary effects. The 'bior3.1' is a typical wavelet function that provides signal decomposition and reconstruction, allowing noise to be isolated while preserving crucial features, and boundary or signal extension mode $M$ set as 'periodic’ defines the handling of signal boundaries during the decomposition process. The output of the 'dwt’ function was stored in array of signal coefficient $(X_{coeff})$. At line 3, mean absolute deviation $(Mean_{dev})$ function was used to estimate the signal’s noise level, whereas, the value $\left( \frac{1}{0.6745}\right)$ matches the standard deviation parameter for Gaussian noise. Following this, a threshold value $(V_{thresh})$ was calculated to determine which coefficients to retain or suppress (i.e., large or small coefficients). The formula $sqrt(2 \times log(len(X)))$ derived from Donoho’s universal threshold formula, was used for optimal noise reduction. At line 6, the 'threshold' function from 'pyWavelet' library was utilized to eliminate small and noisy coefficients by providing list of coefficient ${coeff[k]}$, the threshold value $V_{thresh}$ and threshold mode $(T_{m})$. The threshold mode has typically five types including soft, hard, garrote, greater, less. The resulting coefficients were stored in the final array $(F_{coeff})$. Finally, the function wave reconstruction $(waverec)$ from 'pyWavelet' was used to reconstruct the denoised signal, and the output was passed as the returned signal $(R_{sig})$. In Fig. 2, a comparative analysis of different wavelets is presented. It is evident that denoising at level 2, when combined with the $T_m$ mode using the ’soft’ thresholding method, introduces distortions in the denoised signal, making it unsuitable for classification tasks. Consequently, the ’hard’ thresholding approach at level 1 is preferred for ECG signals, as it effectively removes noise while maintaining signal continuity and avoiding sharp discontinuities. Among the evaluated wavelets, 'bior3.1' demonstrated minimal alterations in the P-wave when compared to the original signal, making it the most suitable choice for this study. In contrast, wavelets such as ‘sym6’, 'bior2.8', and 'db2' exhibited greater deviations and abrupt changes in signal morphology. The 'rbio1.5' wavelet produced results comparable to 'bior3.1'; however, 'bior3.1' better preserved the QRS and T-wave structure, closely matching the original signal’s shape. Unlike typical high low pass filter, the cutoff frequency is not fixed numerically in discrete wavelet transformation that mainly works on principal of signal decomposition and reconstruction. Keeping in the presented situation, where wavelet type was set to bior3.1, decomposition level to 1 with 100 hz sampling rate, the wavelet transform splits the frequency range of the signal (0 to 50 Hz) into two bands. The ‘approximation coefficients’ ranging 0 to 25 Hz and ‘detail coefficients’ with 25 to 50 Hz frequency. Therefore, the approximation coefficients capture 0 to 25 Hz, and the cutoff frequency is $\sim$25 Hz at level 1, presenting boundary between low-frequency (approximation) and high-frequency (detail) components.

The Biosignal processing library in Python (Biosppy) ^27^ is a widely-used open-source tool for processing biomedical signals, including ECG, photoplethysmography (PPG), and electroencephalogram (EEG). It offers stable and reliable functions such as heart rate estimation, peak detection, and signal segmentation. Likewise, the Scipy signal ^28^ library offers various signal processing function including highpass, and digital filters. We realized that the Biosppy library’s R-peak detection could struggle with extreme ECG signals, particularly those with motion artifacts. To address this, we resampled the data with down sampling of frequency using the Scipy library and implemented a Python script that leveraged the Biosppy library for R-peak detection to make the CPSC-2018 dataset comparable with PTB-XL. For the CPSC-2018 dataset, which contains 30 to 60-second long ECG signals, the R-peaks detection was used to divide each signal into three or more 10-second segments. This segmentation allows for a more granular analysis of the heartbeats in smaller intervals, which can improve model training and classification performance. Both of these techniques i.e., resampling and R-peaks aimed at enhancing the data quality and improving the overall model performance, particularly when handling datasets with potential signal inconsistencies. To optimize memory usage and maintain feature quality facilitated by the segmentation process, the final PNG resolution was set to 96x96 instead of high resolution such as 256x256, providing a balance between memory efficiency and preserving essential signal features for model training.

For the CinC-2017 dataset, which provides single-lead ECG signals, the highest peak was detected by finding the amplitude using Scipy highpass, and digital filters with an additional step: the signals were flipped if they showed negative deflection (Algorithm 2). This step was crucial to ensure the signals were aligned correctly for further processing and classification. By flipping the negatively deflected signals, the approach helps to standardize the input data, making it more consistent and better suited for deep learning model training. Skipping the denoising procedure may leave the ambiguous patterns in signals, which makes it difficult for deep learning models to analyze them effectively. Similarly, failing to maintain consistency in intervals, amplitude, and frequency across the three datasets can introduce high variations between the training and testing sets, potentially resulting in poor model performance. Additionally, bypassing signal segmentation would require presenting the input data in high dimensions, which would significantly increase the demand for computational resources.

### Model architecture

**Figure 3:**
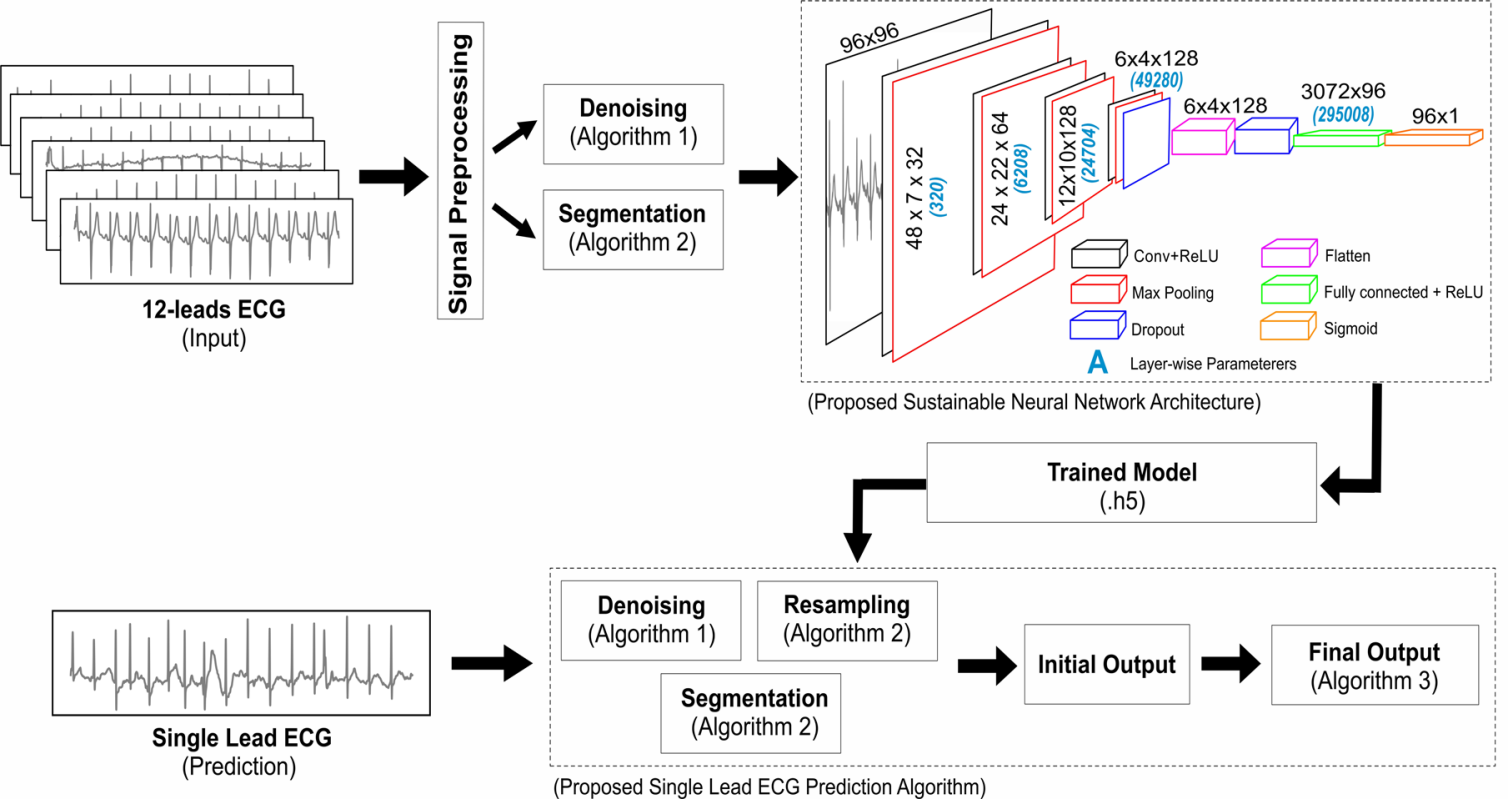
An in-depth analysis is performed on a CNN that is sequentially trained on each lead of a 12-lead clinical ECG dataset, one lead at a time, to predict single-lead ECG on real data. The proposed memory-optimized CNN architecture consists of 32, 64, 128, and 128 filters, various feature map size dimensions of each layer i.e., 48$\times$7, and ‘ReLU’ activation function. The model is trained in conjunction with preprocessing techniques (given in the algorithms) to ensure compatibility and effectiveness with the 12-lead format.

A convolutional neural network (CNN) model, favored in image classification, on Keras and TensorFlow framework was considered. A variety of hyperparameters, such as different numbers of filters and layers, were tested initially. However, these experiments yielded lower classification results. To achieve more robust performance, the final model was simplified and designed as shown in Fig. 3. It consists of four convolutional layers with 32, 64, 128, and 128 filters, respectively. Each layer uses the Rectified Linear Unit (ReLU) activation function, ‘max pooling’ for downsampling, and dropout regularization (ranging from 0.25 to 0.50) to prevent overfitting. ReLU activation function transforms all input values to positive numbers, which helps to reduce computational complexity. This transformation is mathematically expressed by Equation 1.

1 $$\begin{aligned} f(x)_{ReLU}= max(0,x) \end{aligned}$$

The gradient of the ReLU function is 0 when *x* is less than or equal to 0, and equal to *x* when it’s greater than 0. This property makes ReLU computationally efficient and helps avoid issues like vanishing gradients. The pooling layer in a CNN reduces the spatial dimensions of the feature map, which not only helps in reducing computational complexity but also preserves the most important features of the data. This process helps in minimizing overfitting. Typically, there are three types of pooling operations: max pooling, min pooling, and average pooling. These are mathematically represented in equation 2, where average pooling computes the average value of a region, helping to retain essential information while reducing sampling size.

2 $$\begin{aligned} P_\text {max} (\textbf{x})=\max \left\{ x_{i}\right\} _{i=1}^{N} \quad P_\text {min} (\textbf{x})=\min \left\{ x_{i}\right\} _{i=1}^{N} \quad P_\text {avg}(\textbf{x}) = \frac{1}{N}\sum _{i=1}^N {|x_i|} \end{aligned}$$

To further prevent model overfitting, a dropout layer was added following the flatten layer. This layer randomly drops a fraction of the neurons during training, which helps prevent the model from becoming overly reliant on specific neurons. The fully connected layer was designed with 96 filters, enabling the model to capture complex relationships between features. Finally, an output dense layer with a ’sigmoid’ activation function was used to produce the binary classification output. The purpose of the loss function is to quantify the difference between the actual and predicted attribute labels, providing a measure for model optimization. This can be mathematically represented by Equation 3.

3 $$\begin{aligned} J(\theta ) = - \frac{1}{m} \left[ \sum _{i=1}^{m} \sum _{j=0}^{3} 1\{y_i = j\} \log \frac{e^{\theta _j^T x_i}}{\sum _{l=0}^{3} e^{\theta _l^T x_i}} \right] + \frac{\lambda }{2} \Vert \theta \Vert \end{aligned}$$

where *m* represents the total number of training instances, with *i* being the index of each instance. *T* denotes the transpose of the matrix, and *j* indicates the total number of classes. The model learns the parameter vector $\theta$, with $\lambda$ acting as the regularization parameter.

The rationale to use only four layers was made to minimize the consumption of graphical processing memory (GPU memory). To address the discrepancies between the 12-lead clinical ECG data and the SL-ECG, a new architecture incorporating three additional layers was developed. These layers aim to resolve issues related to variations in sampling rate, amplitude, lead deflection, and signal interval.

In the first layer, the ‘resampling’ and ‘normalization’ functions (Algorithm 2) were applied to make the SL-ECG test sets more comparable with the training and validation data from the 12-lead clinical dataset. This enhancement allows the DL model to perform more accurate classifications. The highpass and digital filter was introduced to correct negatively deflected SL-ECG samples, which helps avoid misclassification. The second layer (Algorithm 2) focuses on signal segmentation, offering two primary benefits: reducing the dimensionality of ECG data to conserve memory and processing resources, and facilitating the detection of AFib episodes within each segment. Finally, the third layer (Algorithm 3) implements a ‘voting’ function, which consolidates the labels of different segments into a single, unified decision. This voting average approach boosts model performance by aggregating information from all segments. The details of the challenges addressed by this architecture are discussed in the following section 4.1.

The trainable parameters in the proposed model are distributed as follows: 320 in the first layer, 6208 in the second, 24,704 in the third, 49,280 in the fourth, and 295,008 in the dense layer. This brings the total number of trainable parameters to approximately 375,520. It is essential for both training and testing data to have somewhat equal representation when training a deep learning model. However, discrepancies in frequency and amplitude between the sets arise due to the differing origins of the data, i.e., ECG devices, including standard clinical systems and smart devices, resulting in unequal representation. The proposed CNN model, enhanced with additional prediction techniques, is shown in Fig. 3.

**Algorithm 2:**
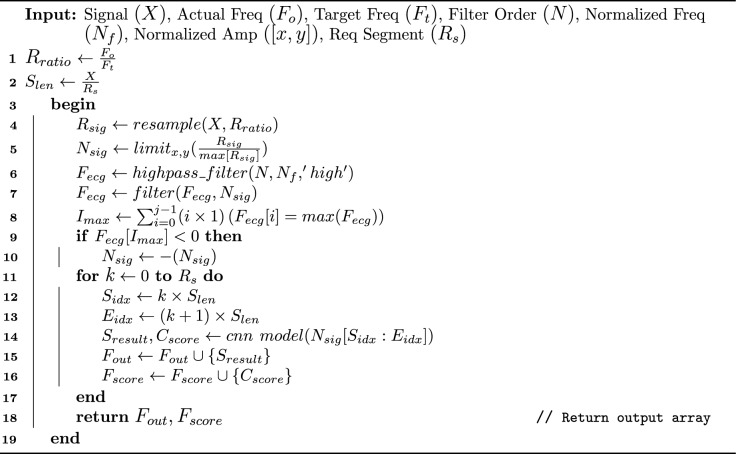
Proposed Single Lead ECG (SL-ECG) Prediction Algorithm

In algorithm 2, several parameters are provided as input. First, the resampling ratio ($$R_{ratio})$$ is calculated, and the segment length $$(S_{len})$$ is computed using a fraction. At line 4, a built-in $$resample$$ function from the sklearn library is given. This function is used to mitigate the frequency differences between the two types of ECG signals, i.e., 12-leads and SL-ECG. The resampled signal is stored in $$(R_{sig})$$. At line 5, signal amplitude normalization is applied to manage the amplitude inconsistencies. The ’clip’ procedure from the Numpy library is used within specified limits $$x,y$$ (e.g., $$[1,-1]$$). The resulting expression returns 1 if $$\frac{R_{sig}}{\max (R_{sig})} > 1$$, returns 0 if $$\frac{R_{sig}}{\max (R_{sig})} < 1$$ and returns $$\frac{R_{sig}}{\max (R_{sig})}$$ otherwise that are used in re-calculating the amplitude without disturbing the actual signal shape. The normalized signal is stored in the variable $$N_{sig}$$.

At line 6-10, the 'highest peak detection’ function using highpass & digital filter is introduced to identify negative lead deflection that could lead to inaccurate data representations, which in turn may reduce the model’s accuracy during training and testing. Built-in functions such as highpass filter $butter$ and additional digital filter $filtfilt$ from Scipy are used for detecting hightest peak and filtering the signal, respectively. The final output is stored in $(F_{ecg})$. At line 8, the Numpy $argmax$ function used to locate the maximum index in the $F_{ecg}$. At line 9-10, $if-else$ statement is employed to compare the maximum index $I_{max}$. If the value is less than 0, it indicates the signal could be negatively deflected that should not be the case in SL-ECG. Therefore, the deflection is reversed to avoid potential misclassification.

At layer 2, the training and validation sets consist of 10-second ECG segments, while the testing set includes 30-second ECG segments. To align the testing data with the training/validation set, the 30-second SL-ECG is segmented into three 10-second fragments. This segmentation provides two key benefits: firstly, it ensures the presentation of all relevant features, and secondly, it reduces memory consumption by working with smaller 96x96 dimensions instead of larger 256x256 dimensions.

Therefore, a loop (at line 11) was executed until the required number of segments $(R_s)$ were processed. For each iteration, the start $(S_{idx})$ and end $(E_{idx})$ indices of the segment were computed. The trained CNN model was then called with the normalized signal $N_{sig}$ and the corresponding segment indices $S_{idx}$ and $E_{idx}$ as inputs. The model output $(S_{result})$ and the associated confidence score $(C_{score})$ for each segment were collected and appended to the final output $(F_{out})$ and confidence score $(F_{score})$, which were then returned.

**Algorithm 3:**
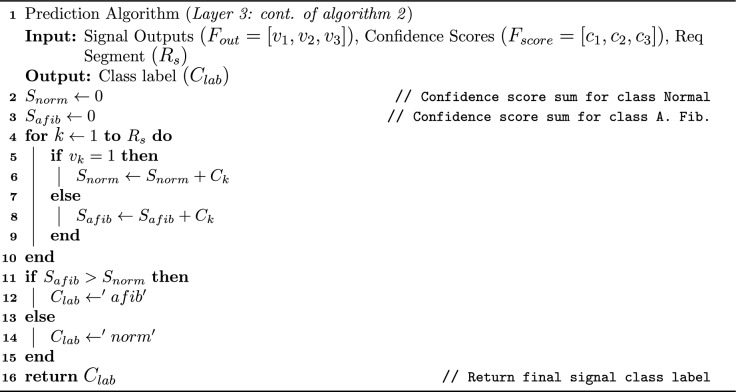
Proposed Voting Mechanism For Single Lead ECG

At layer 3, the model produces three class labels and their corresponding confidence scores for the three 10-second segments. To consolidate these into a single final label for each sample in the test set, a voting mechanism is introduced. If the sum of the confidence scores of two similar labels is less than that of the third, the final label will be chosen from the third segment, as it has the highest confidence score and better detects the AFib episode. In algorithm 3, the output of the prediction algorithm 2 is passed as input, including an array of segment outputs $(F_{out} = [v_1, v_2, v_3] )$and the model confidence scores $(F_{score}=[c_1, c_2, c_3])$ representing the outcome for each segment in the given sample. At line 2, since each sample has three segments with two types of classification labels, two empty variables, $S_{norm}$ and $S_{afib}$ representing normal and disease, are declared to store the class labels. At line 3-9, a $for \ loop$ is used to calculate and store the confidence scores into $S_{norm}$ and $S_{afib}$. At line 10, an $if-else$ statement allocates the final class label $C_{lab}$ as either ‘afib’ or ’norm’, indicating normal or AFib, respectively and return it. The voting average function further enhances the accuracy by about 2 to 3%. The entire framework, shown in Fig. 3, is fully automated, from image receiving to prediction.

### Performance metrics

The evaluation of the proposed model is conducted using standard performance metrics, which are commonly used in existing work and are well-suited for assessing ECG signals and deep learning (DL) models. One of the key metrics is accuracy, which is computed using existing libraries, such as TensorFlow Keras metrics, during the model training and testing phases. Additionally, confusion matrices are generated using the sklearn library to assess the model’s classification performance. Other important metrics, such as sensitivity and specificity, are estimated from the confusion matrix ^29–31^.

4 $$\begin{aligned} \text {Sensitivity} = \frac{\text {True Positives}}{\text {True Positives} + \text {False Negatives}} \end{aligned}$$

The sensitivity, also known as the true positive rate, is given by the Equation 4 and measures the performance of the model by quantifying its ability to correctly identify actual positives in relation to the predictions. In other words, it indicates how many individuals who are actually sick are correctly classified as sick by the model. The higher the sensitivity, the better the model is at identifying true positives, which in the case of ECG signals would correspond to correctly identifying AFib episodes.

5 $$\begin{aligned} \text {Specificity} = \frac{\text {True Negatives}}{\text {True Negatives} + \text {False Positives}} \end{aligned}$$

Similarly, specificity, or the true negative rate, as given in Equation 5, quantifies the actual negative samples in relation to the model’s predictions. In other words, it describes the proportion of normal individuals who are correctly classified as normal by the model. A higher specificity indicates that the model is better at avoiding false positives, thus correctly identifying healthy individuals as not having AFib.

6 $$\begin{aligned} \text {Area Under ROC} \approx \sum _{i=1}^{n-1} \frac{1}{2} (TPR_i + TPR_{i+1}) (FPR_{i+1} - FPR_i) \end{aligned}$$

The other popular methods to evaluate the classification model performance on a test set are the Receiver Operating Characteristic (ROC) curve and the Area Under the Curve (AUC). The equation 6 describes the numerical approximation of the ROC-AUC ^32^ using the trapezoidal rule for all of the samples within the test dataset, i.e., sigma belonging 1 to $n-1$. True Positive Rate (TPR) represents the sensitivity, and False Positive Rate (FPR) can be measured with (False Positive/False Positive+True Negative). The sklearn library ^33^ provides a built-in function to calculate this metric and was used for evaluating the model’s performance.

## Results and discussion

The Jupyter notebook, Keras, and TensorFlow frameworks were configured on Ubuntu 22.04 LTS with an RTX 2070 GPU (14 GB memory). Python was used for the entire experiment. SL-ECG data from smart device were compared with clinical ECG to ensure better classification. The CNN model was trained separately on each 12-lead ECG. Initially, the model was trained on 12-lead data and tested on the CinC-2017 dataset, yielding low accuracy (40-45% on leads I and III). The challenges responsible for this performance drop are discussed in Section 4.1.

### Challenges

The training and testing data came from different ECG devices standard clinical machines and smart devices. As a result, the following challenges were identified to better align the signals for classification:

- The clinical data operated at sampling rate of 100 Hz and 500 Hz, while the smart device data was sampled at 300 Hz, leading to mismatched temporal resolutions.
- A clear difference in signal amplitudes was observed between the two types of devices, complicating direct comparison and classification.
- The smart device ECG signals exhibited negative deflections that should not be the case, leading to potential misinterpretation and classification errors.
- The training set consisted of 10-seconds (PTB-XL) and 30 to 60 seconds (varied in CPSC-2018) ECG segments, whereas the testing set included longer 30-seconds (CinC-2017), adding further complexity in aligning the data for meaningful analysis.

The variation in sampling rate and amplitude differences between clinical and smart device ECG signals posed a challenge for the DL model, leading to reduced performance. To address the sampling rate mismatch, Layer 1 uses the 'resample’ function, while the 'normalization’ function standardizes the amplitude using Numpy’s 'clip’ method. Additionally, the negative deflections found in smart device ECG signals, likely due to improper placement, caused misclassifications, as shown in Fig. 7 (aVR). To mitigate this, Layer 1 includes an 'highest peak detection’ function, which identifies negative deflections and inverts the signals to ensure positive deflections, improving classification accuracy.

The variable ECG signal lengths across datasets, such as PTB-XL, CPSC-2018, and CinC-2017 (10 vs. 30 to 60 seconds or longer), posed challenges for DL model consistency. To standardize the data, long leads were split into 10-second segments for training, while testing on CinC-2017 followed the same approach. Additionally, 10-second leads were represented with a 96x96 image, requiring fewer computational resources, while long interval leads needed larger images, leading to increased resource demand. These variations in data distribution impacted model accuracy. The newly proposed architecture, shown in Fig. 3, addresses these issues, retraining on individual leads and testing on SL-ECG (CinC-2017).

### Decision boundary impact

**Figure 4:**
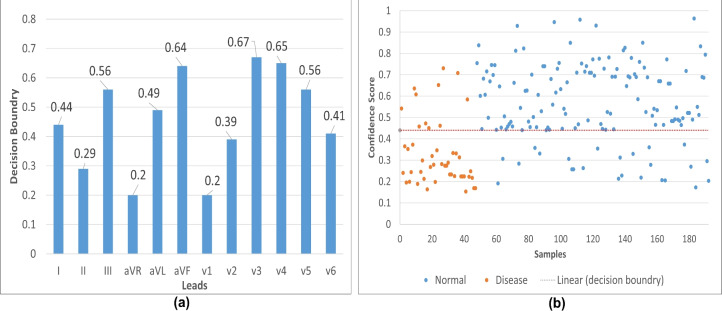
Trained model parameters setting for testing CinC-2017 dataset (**a**) A binary cutoff was selected for each isolated lead trained model (.h5), where the testing performance showed the highest possible accuracy. (**b**) The appearance of single lead ECG (SL-ECG) testing samples was evaluated on the clinical lead I trained model, with decision boundaries tested at various thresholds (i.e., 0.40, 0.44) to optimally separate the classes. The decision boundary refers to a threshold, used to convert the model’s continuous output, i.e., probability score into binary class label.

To evaluate the trained model (.h5 file) of each isolated clinical lead on CinC-2017 validation samples, it was essential to determine an appropriate threshold for the binary classification decision boundary. In the Keras framework, the model outputs values between 0 and 1, with a score near 0 indicating disease and a score near 1 indicating normal (in this case). The critical task was to find the threshold that best separates the two classes. Various boundary values were tested, ranging from 0.50 upwards or downwards, with the final optimal values shown in Fig. 4(a) for each lead model. Although a value close to 0.50 tends to yield the best classification performance, the threshold can vary slightly, but it should avoid extreme low or high values like 0.20 or 0.80. Similarly, Fig. 4(b) showing the linear decision boundary impact when it was set to 0.4 in separating testing samples for Normal and Disease class.

**Figure 5:**
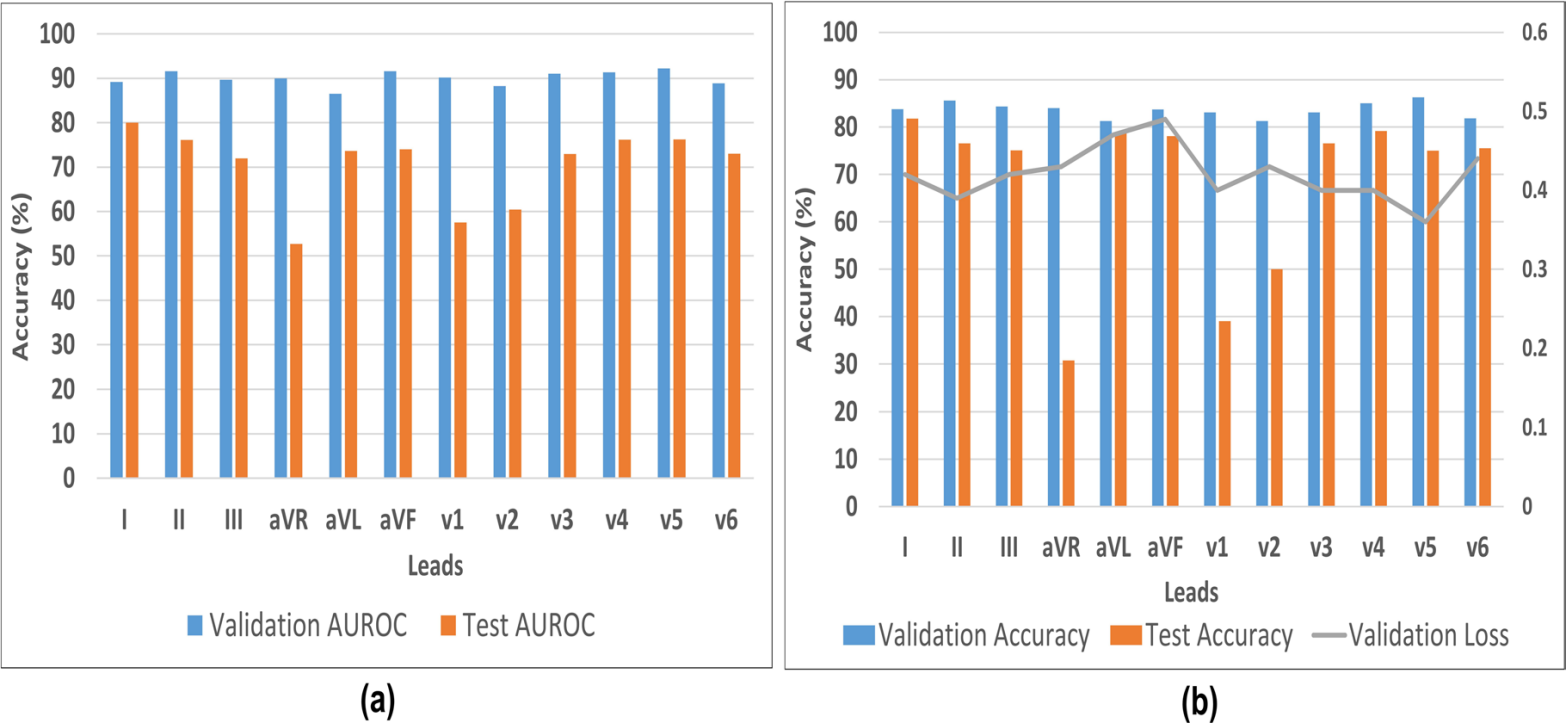
The 2-dimensional model performance evaluation metrics are presented with leads on the x-axis and accuracy (%) on the y-axis. (**a**) The Area Under the ROC Curve (AUROC) for both validation (PTB-XL, CPSC-2018) and testing (CinC-2017) datasets is shown. (**b**) Accuracy and loss curves for the validation (PTB-XL, CPSC-2018) and testing (CinC-2017) sets are provided. A notable observation is that the loss and lead deflection are correlated with testing accuracy. For instance, lead aVR, which exhibits negative deflection during training, resulted in lower testing accuracy for single lead ECG in the CinC-2017 dataset.

Fig. 5(a) illustrates the comparative performance of the Area Under the ROC Curve (AUROC) for each lead during model validation (PTB-XL, CPSC-2018) and testing (CinC-2017) on 12 isolated lead models. The assessment of these models on the unseen CinC-2017 dataset shows that leads I, aVL, aVF, v4, and v5 perform better for smart device signal predictions. The performance differences across leads can primarily be attributed to deflection. Since SL-ECG uses lead I morphology, higher accuracy was observed when the model was trained on lead I. However, lead aVR, which shows negative deflection, performed poorly as SL-ECG is positively deflected. This mismatch caused the model to misclassify SL-ECG samples as disease, resulting in higher predictions for the disease class in lead aVR (given in Fig. 7). In terms of accuracy (given in Fig. 5b), leads I, aVL, aVF, and v4 showed consistent results. Leads with negative or biphasic deflection like aVR, v1, v2, and v3 showed less structural similarity with SL-ECG, leading to decreased model performance. Similarly, clear visibility of P-QRS-T waves without significant noise or baseline drift contributes to improved model accuracy. To ensure cleaner inputs, noise was removed from both the training and test sets prior to model evaluation.

**Figure 6:**
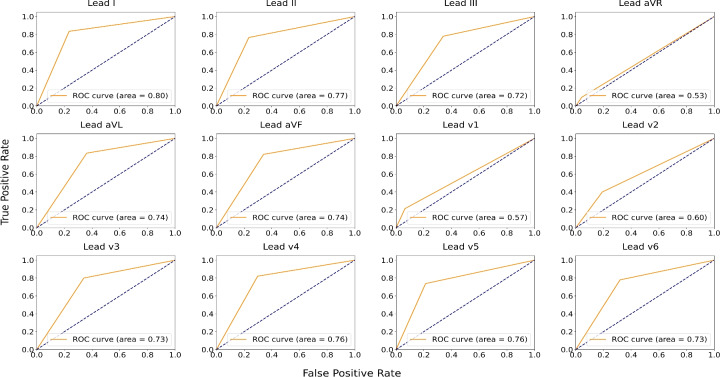
The area under receiver operating characteristic curve obtained from 2-dimensional model trained on 12-leads clinical ECG and tested on smart device oriented single lead ECG.

Fig. 6 presents the area under ROC analysis for each model trained on separate 12-lead ECG data and tested on SL-ECG. Area under ROC, calculated using 'roc_auc_score’ from 'sklearn.metrics’, measures the model’s ability to distinguish between classes, where a higher area under ROC indicates better performance. Notably, leads aVR, V1, and V2 exhibited a comparatively lower area under ROC than the other leads, suggesting a reduced classification performance. This reduction can be attributed to the negative deflection in these leads, which impacts the model’s ability to generalize effectively.

### Time series classification

The 1D-CNN model was trained separately on each of the 12 individual leads and tested on SL-ECG using the proposed approach. The one-dimensional data for each sample was stored in NumPy arrays and divided into training, validation, and testing sets. However, an analysis of the data from PTB-XL, CPSC-2018, and CinC-2017 revealed differences in their NumPy array compositions. To standardize the data across all sets, a Python script was used to apply functions such as transposition, signal resampling, and NumPy clipping (normalization). These preprocessing steps ensured that all datasets maintained a consistent shape, ultimately aiding CNN-based classification. Each lead contained approximately 900 data points per features. The model’s performance is summarized in Table 2.

**Table 2:** Classification performance of 1-dimensional CNN model on time series ECG data.

Leads/Metrics	I	II	III	aVR	aVL	aVF	v1	v2	v3	v4	v5	v6
**Accuracy (%)**	65.11	63.02	52.08	50.1	48.96	51.56	53.12	60.42	40.1	46.35	58.85	62.5
**AUROC**	0.51	0.53	0.42	0.5	0.41	0.47	0.38	0.42	0.41	0.46	0.512	0.5
**Sensitivity**	0.79	0.72	0.61	0.99	0.56	0.56	0.67	0.78	0.39	0.46	0.66	0.74
**Specificity**	0.23	0.36	0.23	0.1	0.28	0.38	0.11	0.06	0.45	0.47	0.36	0.26

One key reason for the reduced performance is the high number of features, i.e., 900 per lead per sample, indeed require deeper CNN architectures to effectively capture relevant patterns. However, this increases computational complexity, which can make training slower and sensitive to overfitting. 1-dimensional CNN model may struggle to capture long-range dependencies in ECG signals, especially when each lead is treated individually without leveraging spatial relationships. If the filters do not extract meaningful features across the entire sequence, classification performance may drop. Additionally, few leads, such as aVR, exhibited misleading classification, where the trained model misclassified all disease samples while correctly identifying normal ones. This resulted in high sensitivity but extremely low specificity.

While feature reduction techniques like principal component analysis (PCA) could be considered, they increase the risk of losing important features. To avoid this, such approaches were not applied. Converting ECG signals into 2-dimensions (i.e., images) allows the model to enhance spatial feature extraction, making pattern recognition more effective, since, CNN models are considered proficient in such tasks. The results clearly indicate that classifying ECG signals using time-series data requires a more complex neural network architecture. Given these challenges, we explored a less complex CNN model using two-dimensional data (PNG images), which yielded better accuracy, as shown in Table 3.

### Two-dimensional classification

The proposed model, shown in Fig. 3, was trained on isolated clinical leads and tested on unseen samples from the CinC-2017 dataset. Instead of using 1-dimensional signals, the ECG waveforms were converted into low-resolution grayscale PNG images, which were then fed into the model. The testing process was fully automated, from generating images to classification, minimizing manual effort and smooth the model pipeline.

**Figure 7:**
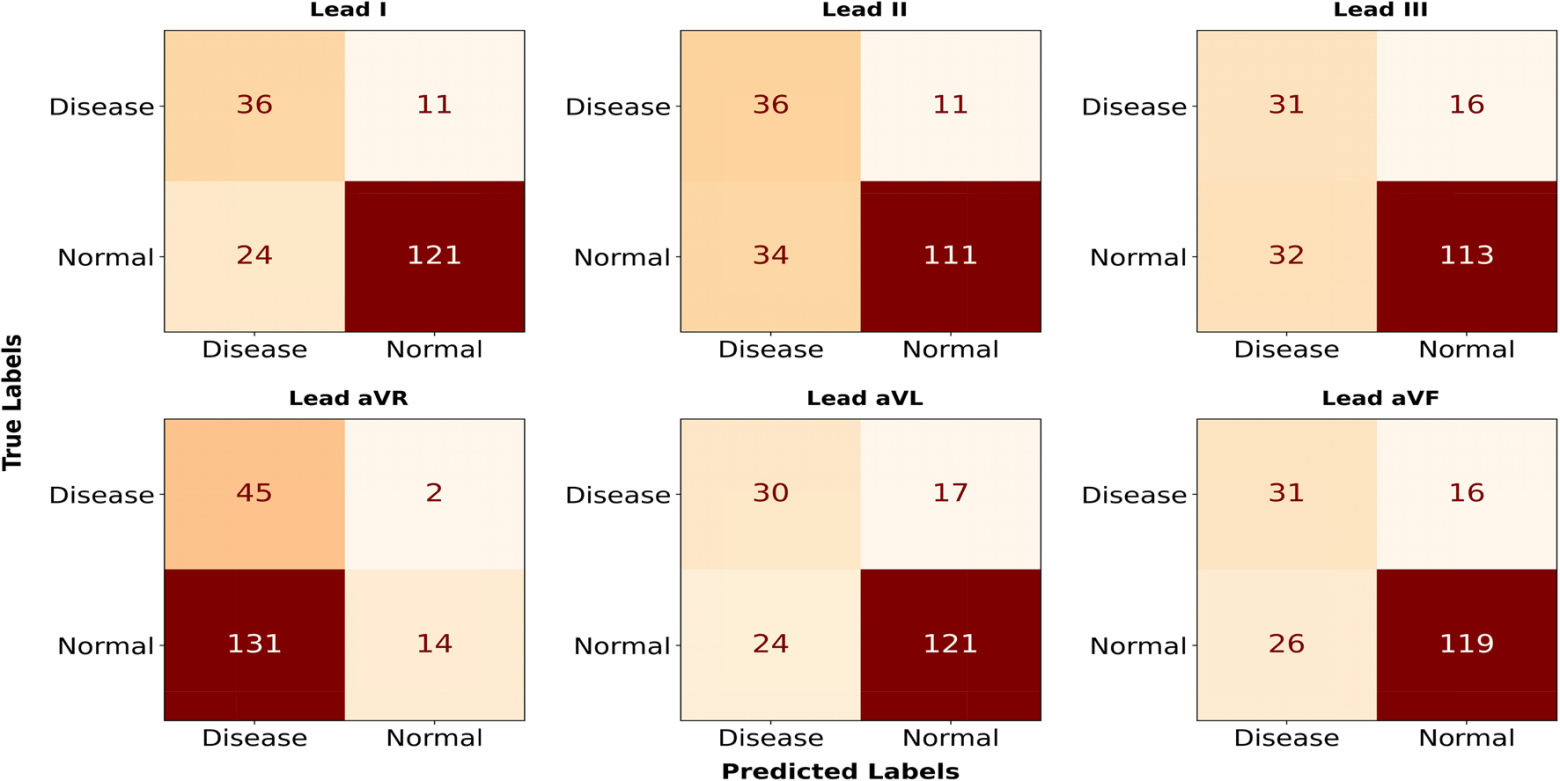
The confusion matrices illustrate classification results across limbs (I, II, III) and augmented (aVR, aVL, aVF) ECG leads, where the 2-dimensional model is trained on individual leads and tested on SL-ECG. Lead I, that align well with single lead ECG morphology, yielding higher accuracy.

The confusion matrix, generated using the sklearn library, highlights classification outcomes for normal and AFib classes. Leads I, aVL, v5, and v6 demonstrated superior performance, with lead I showing the highest correctly classified samples. This is attributed to their structural similarity to SL-ECG. A slightly lower accuracy in v5 is observed, while aVL and v6 maintain comparable results. The orientation of 12-lead ECG angles and their relation to SL-ECG can be further explored in our previous work ^15^.

Leads II, III, and aVF show comparable results, unlike aVR, which performs poorly due to its negative deflection. Since SL-ECG signals are typically positively deflected, this mismatch disrupts proper data distribution in the DL model. As a result, the model misinterprets negative deflections as abnormal, leading to higher disease classifications in the aVR confusion matrix (Fig. 7).

**Figure 8:**
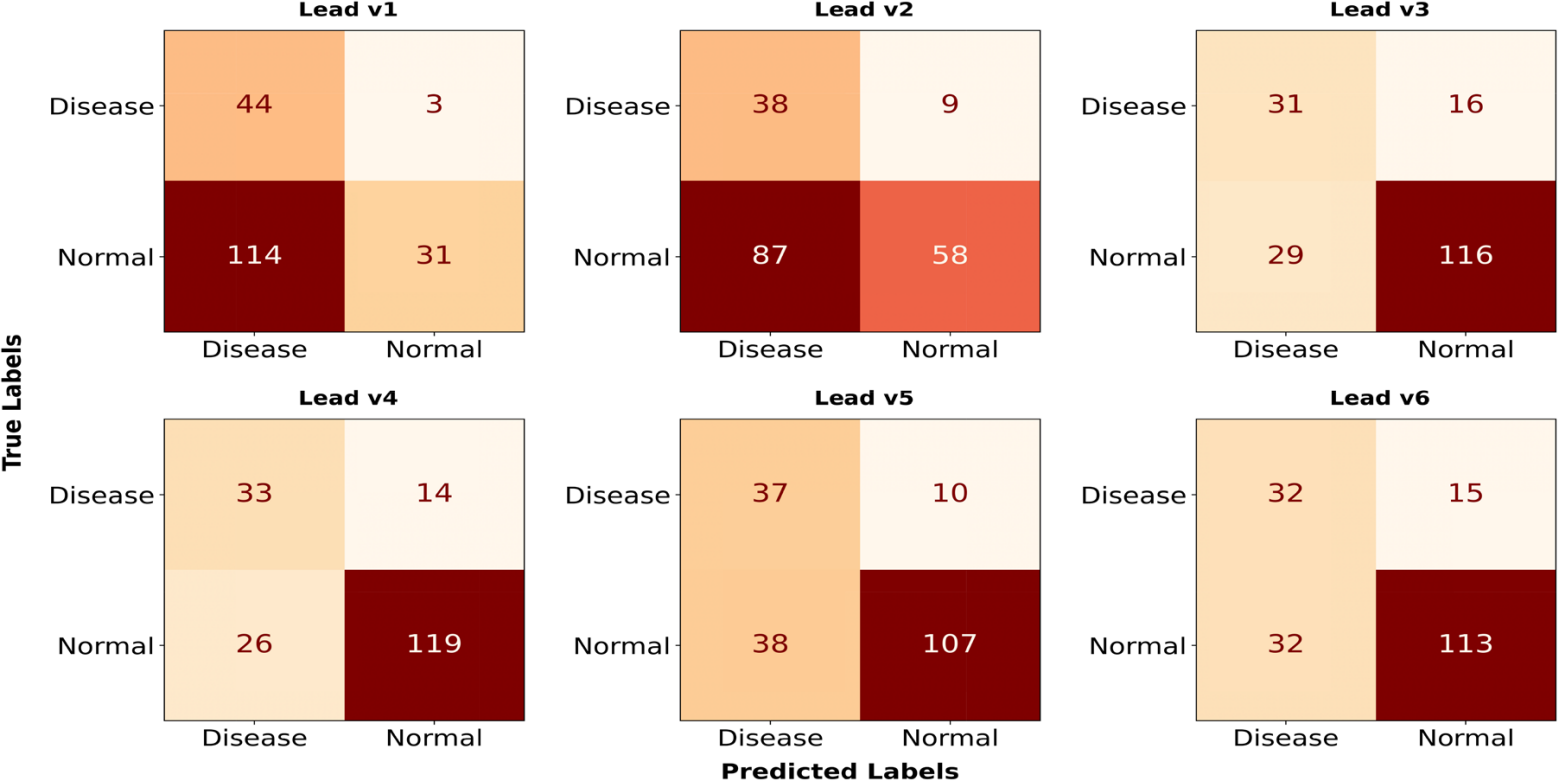
Confusion matrices (2-dimensional model) for six chest leads, highlighting differences in signal deflection. Leads v1-v2 and v3-v4 may exhibit biphasic or slightly negative deflection, whereas v5 and v6 tend to align more closely with the single lead ECG morphology, impacting classification performance.

In a similar context, leads v1 and v2 exhibit negative or biphasic deflection, reducing their structural similarity to SL-ECG. The confusion matrix shows a pattern similar to lead aVR, where the model frequently predicts disease. However, as the deflection shifts from negative to positive in leads v3 to v6, the classification improves. Consequently, leads v3 and v4 demonstrates slightly better performance, comparable to v5 and v6 (shown in Fig. 8).

**Table 3:** Performance of isolated clinical lead wise trained 2-dimensional CNN model on CinC-2017 test set.

Leads/Metrics	I	II	III	aVR	aVL	aVF	v1	v2	v3	v4	v5	v6
**Accuracy (%)**	81.77	76.56	75.1	30.73	78.65	78.12	39.06	50.2	76.56	79.17	75.3	75.52
**AUROC**	0.81	0.76	0.71	0.52	0.73	0.74	0.57	0.6	0.72	0.76	0.76	0.73
**Sensitivity (%)**	76.6	76.59	65.95	95.74	63.82	65.95	93.61	80.85	65.95	70.21	78.72	68.08
**Specificity (%)**	83.44	76.55	77.93	0.09	83.44	9.61	21.37	40.01	80.2	82.06	73.79	77.93

Table 3 presents the performance of each isolated clinical lead-trained CNN model, tested on smart device oriented CinC-2017 samples, including sensitivity and specificity. Lead I shows the highest efficiency with 76.60% sensitivity and 83.44% specificity. The table highlights the effect of lead deflections on model interpretation, reflected in the sensitivity and specificity values. Overall, leads I, II, v4, and v5 produce a well-generalized model for SL-ECG prediction, with both sensitivity and specificity above 70%.

### Comparison and analysis

The highlighted work introduces a novel approach in terms of its concept, disease target, and the techniques used to improve performance. In the latest review (2022-2024) in Section 2, a few studies were identified, but the presented study differs due to its unique focus. Nevertheless, a comparison has been made based on SL-ECG considerations, as shown in Table 4.

**Table 4:** Performance comparison with prior clinical leads and single lead ECG.

Description		Accuracy	AUROC	Sensitivity	Specificity	Type of Input Signal
Luongo G. et al. ^19^		73.50%	-	64.70%	91.40%	Train (SL-ECG), Predict (SL-ECG)
Jiwoong K. et al. ^20^		70.50%	0.79	79.30%	-	Train (SL-ECG), Predict (SL-ECG)
Lee K.H. et al. ^34^		73.80%	0.79	65.50%	-	Train (SL-ECG), Predict (SL-ECG)
**Proposed Work**		81.77%	0.81	77%	83.44%	Train (12-leads), Predict (SL-ECG)

Previous work by the authors in ^15,35,36^ prompted further investigation into the DL model’s capability for classifying single-lead ECG signals derived from clinical ECG data. Studies by Luongo G. et al. ^19^ and Jiwoong K. et al. ^20^ also focused on binary classification for AFib vs. normal using SL-ECG data, however, our proposed model outperforming both in terms of accuracy and AUROC. Similarly, Datta S. et al. ^21^ addressed the same disease and SL-ECG signals, but due to the absence of key metrics like accuracy, AUROC, sensitivity, and specificity, a direct comparison is not possible. Additionally, their data was acquired exclusively from a single device, while this study utilizes data from both clinical and smart devices, making the classification more challenging. In the work by Khunte A. et al. ^22^, noise-adding techniques were employed for data augmentation, but their lower specificity (52.3%) and higher sensitivity (95.6%) suggest the model needs further generalization for binary classification. Lee K.H. et al. ^34^ initially achieved lower metrics (67.5% accuracy, 64.2% sensitivity), but after applying transfer learning on 12-lead data, results improved to 73.8% accuracy, 65.5% sensitivity. Attia Z. et al. ^23^ used a digital stethoscope for detecting LVSD with single-lead ECG, introducing additional device variability that may introduce bias in the interpretation.

The concern has also raised whether the trained model has ability to distinguish other type of arrhythmia. Deep learning models are limited to classifying the types of data they were trained on. In the current case, the CNN model was trained exclusively on samples labeled as AF and Normal. As a result, it performs well in distinguishing AF from normal rhythms in real-world scenario. However, when the other types of arrhythmia or cardiac conditions will be presented to the model, it will classify labeling them as AF, unless these conditions clearly separate them feature wise from normal signals. This is because the model has only learned to recognize two classes including AF and Normal. Apart from this, transfer learning, an established approach in artificial intelligence can be applied. By re-training the existing model on additional data representing other arrhythmia or cardiac diseases, the model can be adapted to new classification tasks. Scientific evidence supports this strategy, showing promising results even when the additional data is limited in size.

In the existing literature, researchers have been focusing on collecting SL-ECG data generated by various portable devices for training DL models (e.g., ^19,20,22^), a process that is both time-consuming and ethically demanding. Moreover, data from portable devices is not widely available in public repositories like PhysioNet, with only a few small datasets that are insufficient for comprehensive DL model training. In contrast, the Physio-net library offers a sufficient clinical ECG data, such as the PTB-XL dataset, which presents challenges like differing frequencies and intervals when compared to SL-ECG. This situation presents an opportunity to propose a novel framework that leverages clinical ECG data for SL-ECG prediction, addressing the technical issues while allowing for the development of a more generalized ECG predictive model compared to using self-collected portable device datasets.

Portable devices like smartwatches have become valuable tools in remote health monitoring, primarily for identifying individuals who may need medical care rather than diagnosing specific diseases. However, hospitals still depend on traditional ECG machines for thorough heart health evaluations. The proposed framework enhances smart device functionality by integrating SL-ECG classification capabilities, providing timely alerts to patients about the need for hospital visits and assisting clinicians in mitigating heart abnormality risks through early detection. Additionally, the proposed four-layer DL model features a streamlined structure, reducing computational resource requirements, making it ideal for integration into smart devices. The three auxiliary layers can also be adapted for use with PPG signals, further aiding in heart condition assessment.

Additionally, to evaluate model size and training efficiency, we focused on studies that used CNN architectures for ECG signal classification. Two existing models including Elyamani et al. ^37^ and Caesarendra et al. ^38^ were reconstructed and tested alongside proposed model. All three models were assessed under identical conditions: using an RTX 2070 GPU with 14 GB of RAM, consistent batch and input sizes, and the same training and testing datasets. This setup ensured a fair comparison in terms of processing time, accuracy, and parameter count. Our proposed model required approximately 2.56 MB of size, contained 0.37 million parameters, and reached its highest possible performance with an accuracy of 81% taking 6 minutes using early stopping. In comparison, the model by Elyamani et al. ^37^ had a size of 8.64 MB with 2.26 million parameters, taking 16 minutes to achieve an accuracy of approximately 80.09%. Caesarendra et al. ^38^ was considerably larger at 56.89 MB, with 14.9 million parameters, and required 14 minutes to reach an accuracy of 79.55%.

Nevertheless, the model needs an intermediate mini computational resource prior to being deployed directly on edge devices. This is due to its design, which involves continuous analysis of long-term signals, an approach that demands a certain amount of memory and processing power. Edge devices typically have significantly limited computational power. A practical example of such an intermediate device is the Nvidia Jetson Nano board, as demonstrated in the study by Mohebbanaaz et al. ^39^, where a Bidirectional Long Short-term memory (LSTM) model was trained on the MIT-BIH dataset and deployed, achieving 99% accuracy. Alternatively, a CPU-based system with sufficient memory can be used to perform image-based computational analysis. In such a way, data is transmitted from the edge device to the intermediate processor, and the results are then displayed back to the user end. The results also highlighted that the relationship between parameter count and training time is not strictly linear. While models with fewer parameters often train faster, this is also affected by factors such as architectural complexity, skip connections, the number of internal operations, and the optimization strategy used. For instance, Elyamani et al. model took longer to train despite having fewer parameters than Caesarendra et al., possibly due to less efficient architectural design. Overall, parameter-efficient models not only reduce training time and memory usage but also support more resource-conscious AI development.

## Conclusion

This study aimed to identify the most effective leads from 12-lead ECG signals by evaluating the predictive performance of single-lead ECG using deep learning models trained on isolated 12-lead clinical ECG. Key factors contributing to reduced accuracy were highlighted, including variations in sampling rate, amplitude, signal deflection, and interval differences between training and testing datasets. To address these challenges, the presented study introduced sequential neural network model with three auxiliary translational layers, effectively improving classification performance. The ROC analysis suggest that clinical ECG leads such as I, II, v4, and v5 are most suitable for training deep learning models when making predictions on SL-ECG. Additionally, the impact of negatively and biphasically deflected leads, including aVR, V1, and V2, on SL-ECG classification was analyzed using confusion matrices. The proposed framework offers valuable insights for smart device manufacturers incorporating SL-ECG classification with sustainable neural network. It can provide timely alerts to patients about potential hospital visits and assist clinicians in early detection, thereby reducing the risks associated with heart abnormalities. Future work will focus on expanding both clinical and SL-ECG datasets with additional cardiac conditions and assessing transfer learning to enhance performance. Furthermore, precise detection of P-QRS-T waves will be focused, as this would enable machine learning models to classify ECG signals with more transparency and make their outputs more interpretable and trustworthy for physicians.

### Study limitations

The current study has some limitations that need to be addressed for further improvement. First, the amount of data sufficient for optimal training, particularly regarding disease classification (AFib), remains unclear and needs further investigation. Second, the study does not explore the potential benefits of a transfer learning approach, where models trained on 12-lead ECG data could be retrained on SL-ECG data to potentially improve accuracy. Additionally, the performance of negative deflected leads, such as aVR, was not reassessed by taking the inverse of the lead to correct for deflection, which could have enhanced the model performance.

## Data Availability

Data used in this study is available publicly at PhysioNet platform. The software, developed in Python, is available freely on https://github.com/mfarhan166/clinical_sl_ecg/ under MIT license (accessed on 15-September-2025).
